# Pioneering neurohackers: between egocentric human enhancement and altruistic sacrifice

**DOI:** 10.3389/fnins.2023.1188066

**Published:** 2023-10-25

**Authors:** Günter Seyfried, Sandra Youssef, Markus Schmidt

**Affiliations:** Biofaction KG, Vienna, Austria

**Keywords:** neurotechnology, hackers, human enhancement, ethics, technology assessment, do-it-yourself, cyborg

## Abstract

The growing field of neurotechnology (NT) is becoming more and more accessible in terms of reduced costs, increasing availability and reliability of materials, and ways to implant devices. As in other engineering fields such as bio-or information technology, there is a growing community of pioneering hackers who (self-)experiment with NT and develop novel applications. While most debates about NT, its goals and ethical ramifications are usually conducted by professionals in the field (neuroscientists, −engineers, −ethicists), little is known within these institutional frameworks about the motivations, goals and visions of neurohackers and how they view ethical ramifications of NT therapeutics vs. human enhancement. In this study we draw on qualitative interviews with 13 of these neurohacking pioneers, who are interacting with NT from a grassroots perspective (i.e., a bottom-up and community/subculture-oriented approach), and shed light on: how they understand themselves in the context of human enhancement; what the role of invasive NTs is when it comes to identifying as a cyborg; if their practices show a clear distinction between therapy and enhancement; whether human enhancement is always about performance, optimization and functionality; and to which extent neurohackers contribute to “mainstreaming” NT.

## 1. Introduction

The increasing technologization of all aspects of life certainly does not stop with the human body. There is a growing number of people, who are involved in a number of research and development activities in the field of neurotechnologies (NT), inside and outside the academic and institutional context, to build such devices for themselves or others. Whole new contexts of utilization are being created, often with the aim to make life easier and better, but also to explore the potentiality of NT enhanced human body and mind (Teunisse et al., 2019). Human enhancement, sometimes called human augmentation, has increasingly become a topic of interest at the intersection of biological, genetic, neurological and technological innovations and advancements, also sparking a wave of attention by regulators, policy makers, commercial and grassroots interests. It is, however, equally clear that NT are—for the time being—risky technologies, with medical, technological, legal, economic, and ethical challenges. Technical progress intensifies these challenges, as well as the opportunities.

Scientific and engineering advances make NT more and more accessible, not only for medical applications such as prostheses (Dubljevic et al., 2014; Wexler, 2016), but also for non-medical, even do-it-yourself (DIY) use (Wexler, 2016; 2017) and human enhancement (Egner and Gruzelier, 2003; Fregni et al., 2005; Dockery et al., 2009; Meinzer et al., 2014). The reduction in cost and increased availability of materials, software and ways to implant devices into the human body makes NT more accessible for the do-it-yourself (DIY) community and neurohackers (Wexler, 2016; Teunisse et al., 2019). While some researchers have attempted to engage with NT user groups to improve, e.g., BCI tools (Stahl et al., 2017) the DIY community is different from user groups as they have their own (research) goals. A debate about goals, motivation, and possible ethical aspects of NT has so far mostly been conducted among neuroscientists, neuroengineers, neurocompanies, neuroethicists, and policy makers (Bard et al., 2018; Garden and Winickoff, 2018; Ienca et al., 2018; Illes et al., 2019; Josh, 2021; Pfotenhauer et al., 2021; Hyun et al., 2022; OECD, 2022). But what do the growing community of do-it-yourself (DIY) and neurohackers think about NT (Wexler, 2017; Teunisse et al., 2019)? To explore this overarching question, we wanted to address the following five research questions:

1. How do neurohackers understand themselves in the context of human enhancement?
2. How important are invasive NT applications for neurohackers, compared to non-invasive ones?
3. Do neurohacker practices show a clear distinction between therapy and enhancement?
4. From the point of view of the neurohackers: Is human enhancement always about performance, optimization, and functionality?
5. Are neurohackers part of or contributing to “mainstreaming” NTs?

As there is only a limited number of position papers and other written material from neurohackers available, we decided to obtain the necessary information through a series of qualitative interviews with neurohackers themselves. This approach follows the successful method deployed in our previous paper about biohackers (Seyfried et al., 2014).

## 2. Materials and methods

This article builds partly on prior work focusing on an extensive survey of peer-reviewed papers, forum posts, online videos, books, blog posts, portfolios, and news articles, leading to the identification of 56 neurotechnology applications outside of the medical realm (Teunisse et al., 2019). That overview served as a springboard for qualitative research delving further into motivations, goals, financial, and technological practicalities of NT practitioners. We used a combination of in-depth qualitative interviews and content analysis as systematic and empirical formats to ground our study.

Paralleling the broad range of technologies and applications that fall under the umbrella of NT, we followed a wide approach for neurohacking as well, including nootropics (a class of drugs and supplements frequently marketed as cognitive enhancers), deep brain stimulation (DBS), transcranial magnetic stimulation (TMS), transcranial direct current stimulation (tDCS), haptic feedback devices, RFID chips, magnetic implants, implanted sensors and computers, brain computer interfaces, and myoelectric prostheses.

For the purposes of this study, we looked for “neurohackers,” i.e., bodyhackers who make use of NT in their hacking endeavors. By necessity, those practitioners we sought out and contacted were individuals who have published their work, or aspects of it, in internet media outlets and who therefore maintained (albeit varying levels of) visibility in their respective communities. We used our prior research (Teunisse et al., 2019), current literature such as magazine articles (e.g., Spyrka, 2019), as well as Google and DuckDuckGo search engines using keywords such as “biohacking,” “neurohacking,” “open prosthetics,” “chip implants” and “bodyhx” to identify 65 neurohackers and their social media accounts. We then used their social media activities and communities to identify other practitioners through snowball sampling.

We reached out repeatedly to these 65 neurohackers (100%) via email, Twitter, and their websites, receiving 20 responses (30.8%) to our interview requests. Eventually, we obtained 13 interviews (20%) in different formats (see also Table 1): 10 were conducted actively using videoconferencing; one relied on using an online questionnaire format; using Google Forms; and another one was conducted via Email, sending a text file to fill out, as two interviewees preferred to answer in writing. Finally, one interview was extracted from an extensive publication provided by one interview partner. While said publication covered many of the questions we sought to answer, there are some questions that could not be answered—and, indeed, some questions were not answered by all of our 12 “traditional” interviews.

**Table 1:** Age distribution of respondents who contributed to this study.

Age group	Number of participants in age group
30–40	3
40–50	8
50–60	1
60+	1

The interviews were based on a systematic interview guide containing a total of 41 questions (see Supplementary material for complete list of questions), aimed to cover as many key areas as feasible and arranged thematically into six:

1. *Background and entry point*: relevant background information about the interviewees, as well as access points and initial motivations.
2. *Equipment and methods:* which technology each neurohacker has access to and uses, as well as how they got access to it.
3. *Motivation and identity*: exploring how each neurohacker identifies, how and whether identity aspects or visions are shared in a community, and whether there are common associations connected to neurohacking.
4. *Networks and structures:* whether and which activities take place in a group context, whether the interviewees work alone or not, as well as to find out more about practicalities, including feedback, peer review, and financial aspects.
5. *Ethics and risks*: how ethics and safety are perceived and discussed among neurohackers. They were also asked to discuss potential social consequences their activities might bring in general, and for disabled people specifically.
6. *Future outlook*: perspectives on future development, in terms of their own work and practice, as well as the NT field in general.

The interviews were carried out between June 2020 and December 2020. Those interviews carried out in person, via Zoom, had an average length of around 70 min. Interview lengths varied between 25 min and 2 h, were recorded with the permission of the interviewees and transcribed.

Interviewees were assured that their answers would be pseudonymized to reduce the risk of direct assignment of certain statements to anyone in particular. Given their online presences, which would allow our interview partners to be found with relative ease, as well as the fact that gender distribution is not equal in the NT communities or in our interviewee pool and thus, would serve as another identifier. Therefore, gender will not be attributed in this paper beyond disclosing that we worked with nine male and four female interview partners. Throughout this text, in addition to gender-neutral pseudonyms, the singular “they” will be used where pronouns are called for (for age of participants see Table 1).

All interview data was organized and subsequently analyzed using methods of qualitative content analysis. Specifically, units of analysis—sometimes referred to as “quotations”—such as sentences, phrases or even single words are thematic units, which are coded for general and, where applicable, specific categories. Given the nature of our study, we chose to rely on an inductive coding process (Christians and Carey, 1989), which adapts to the data at hand. In other words, codes are developed and adapted based on interviewee’s answers and refined in an iterative process. Similar categories were then clustered into larger thematic topics, if and where patterns emerge. All three authors worked on the coding and analysis process, frequently reviewing, discussing, and cross-checking codes and categories in order to ensure reliability and consistency across all 13 interviews. Since a neurohacker’s answer to one open-ended question can consist of several smaller snippets or phrases that can fall into multiple categories, this approach allows a more nuanced analysis. For the assessment of frequency of codes and categories, it is not relevant how little or much a neurohacker speaks on that theme, merely that it is important enough to mention. In return, the depth of qualitative answers is drawn on for citations that serve to illustrate as well as illuminate the emergent themes. This approach allows the analysis to center around the main protagonists of this text: the neurohackers.

## 3. Results

### 3.1. Background of neurohackers

As pointed out previously in section 2, our interview partners (see Table 2) come from a variety of backgrounds, though the majority, i.e., nine out of 13 people (69%), worked with neurotechnology at least in part in a professional capacity: four as researchers either academically or at a company; two as CEOs of neurotech start-up companies; two in their artistic work and performances; and one respondent (the designer/lecturer) holds a leading position in a cyborg interest group and researches/works in cyborg communities. The remaining four interviewees practice neurotechnology for primarily personal, and non-professional purposes.

**Table 2:** Overview of neurohacker respondents who contributed to this study.

Nick-name	Occupation/day job, cyborg activism, community, bio(logy) background	Location (practice)	Neurohacker since	Interview source
Kee	Contemporary artist, cyborg activist	Worldwide	2004	Sent detailed publication
Pea	Spokesperson for biohacking collective, CEO of NT start-up, Ph.D. Mathematics and Physics, human rights activist, community biohacker	USA and Singapore	2017/18	Video interview
Cal	Performer, cyborg activist, stunt performer	USA	2017	Video interview
Nas	Creative strategist at NT start-up, Ph.D. student (design)	UK	2016	Video interview
Sto	Biologist, founder and researcher of biotech non-profit organization, community biohacker	USA	2010	Video interview
Tao	Founder and CEO of NT start-up, human rights activist	UK	2016	Video interview
Gil	Researcher at an entertainment company	Japan	2017	Email interview
Ari	Digital media and web development, podcaster, community manager, cyborg activist	Germany	2017	Video interview
Rhi	Designer, lecturer, president of a cyborg interest group [Note: Rhi does not practice bio-or neurohacking, but has much exposure to communities and practitioners.]	Germany	Not applicable	Online survey
Sal	Professor emeritus, researcher	UK	1998	Video interview
Vel	Locksmith, community biohacker	USA	2012 (with nootropics 2008/09)	Video interview
Nol	Security guard, community biohacker	Netherlands	General hacker 2001, neuro-hacking 2010	Video interview
Tyr	Salesperson	Austria	2017	Video interview

Did individual education pathways or occupations contributed to or facilitated interviewees’ neurohacking practices (Q4)? The answers to this were not uniform: of 12 respondents to this question, seven saw a connection between their education and neurohacking work, four of those unequivocally and three to some degree. The remaining five respondents saw no connection between their education and their neurohacking.

Our interview partners have come to neurohacking at different times and via different “gateway” technologies (discussed further below), from as early as 1998 to as recently as 2017/18 (Q2). Despite the fact that these entry points span two decades, all of the neurohackers we interviewed can be categorized as either “innovators” or “early adopters” (Rogers, 2003). Typically, the relationship between companies and early adopters is synergistic, which is the case for all our interview partners, who may receive early or unique access to products and in turn may provide companies or providers with crucial feedback.

### 3.2. Technologies and identity

#### 3.2.1. Technologies

In regard to technologies used, the interviews were structured in a way to highlight several areas of interest, including what gateway technologies the neurohackers encountered and accessed first (Q2–3), what technologies they use presently (Q5–6), and how involved each interviewee is with the technologies they use, i.e., how much they “tinker” (Q7). For almost all our interview partners RFID (radio-frequency identification) chips and magnetic implants served as a starting point to go deeper and learn more, representing the most common gateway technology for neurohacking (see also Emporium, 2017; Robertson, 2017). Transcranial direct current stimulation (tDCS) and nootropics are the second most important technologies as a gateway technology in our study (see also Teunisse et al., 2019) for use case examples (TotaltDCS, 2022), and a Reddit thread on transcranial Direct Current Stimulation (Ohsnapitsnathan, 2016) for user discussions and activities including requests on how to translate scientific findings (Zeng et al., 2020) into practical use. Nootropics, although, similarly to other NTs, impact on and benefit to healthy consumers is debated (Esposito et al., 2021). Three of our interviewed neurohackers came to their neurohacking practice through tDCS, electric stimuli and nootropics.

Along with RFID and magnetic implants, four neurohackers started to work fairly quickly toward eventually implementing more invasive and/or complex technologies, including an implant that functions as a wireless router and hard drive, a device, which allows users to sense when they are facing North, an implantable microelectrode array and artificial synesthesia electronics.

Our data shows that the vast majority came in for the technology, be they RFID/NFC chips or magnetic implants. The main sources for NT equipment are professional manufacturers and NT startups, although self-assembled devices from parts off the shelf have been used on a similar scale. Unsurprisingly, given the nature of biohacking and neurohacking activities, a large majority of our interview partners (nine of twelve) have tinkered with NTs in different ways, including low-level experimenting with parameters and setups, building hardware from scratch, programming software, interfacing with other devices (e.g., setups using the Arduino platform), and mixing and testing implant surface coatings.

#### 3.2.2. Motivation

When considering motivation, one point brought up by several interview partners provided the backdrop to when and how they began their neurohacking activities: the role of show-and-tell. Charismatic presentations by practitioners at lectures and talks, along with demonstrations at events were mentioned by several interview partners specifically as contributing motivational forces. <Nas> mentioned for instance “seeing Neil Harbisson, with his talks and videos, seeing the potential and impact and the creativity and innovation that opens up.” <Cal>’s daughter showed them a video of a gamer girl unlocking a computer with a chip and <Cal>, as they put it, “bought on.” <Ari> knew about the existence of implants, but discovered a deeper interest during a lecture held by the founder of an international biohacking platform. They took the opportunity to ask questions on how to go about experimenting with implants.

In this sense, presentations and demonstrations seem to enhance the acceptance of new NT and encourage emulation at least in audience members who may already be interested or predisposed.

In general, these early developers and adopters are highly interested in complex systems, have a strong desire to advance the human condition and a strong affinity to sci-fi literature and media. Several interview partners, for example, mentioned the fictional character “Molly Millions” from William Gibson’s cyberpunk books (e.g., Gibson, 1986) as a role model or example of a cyborg they found inspiring, and others explained they liked sci fi, when talking about their curiosity and openness to neurohacking or implants.

**Figure 1:**
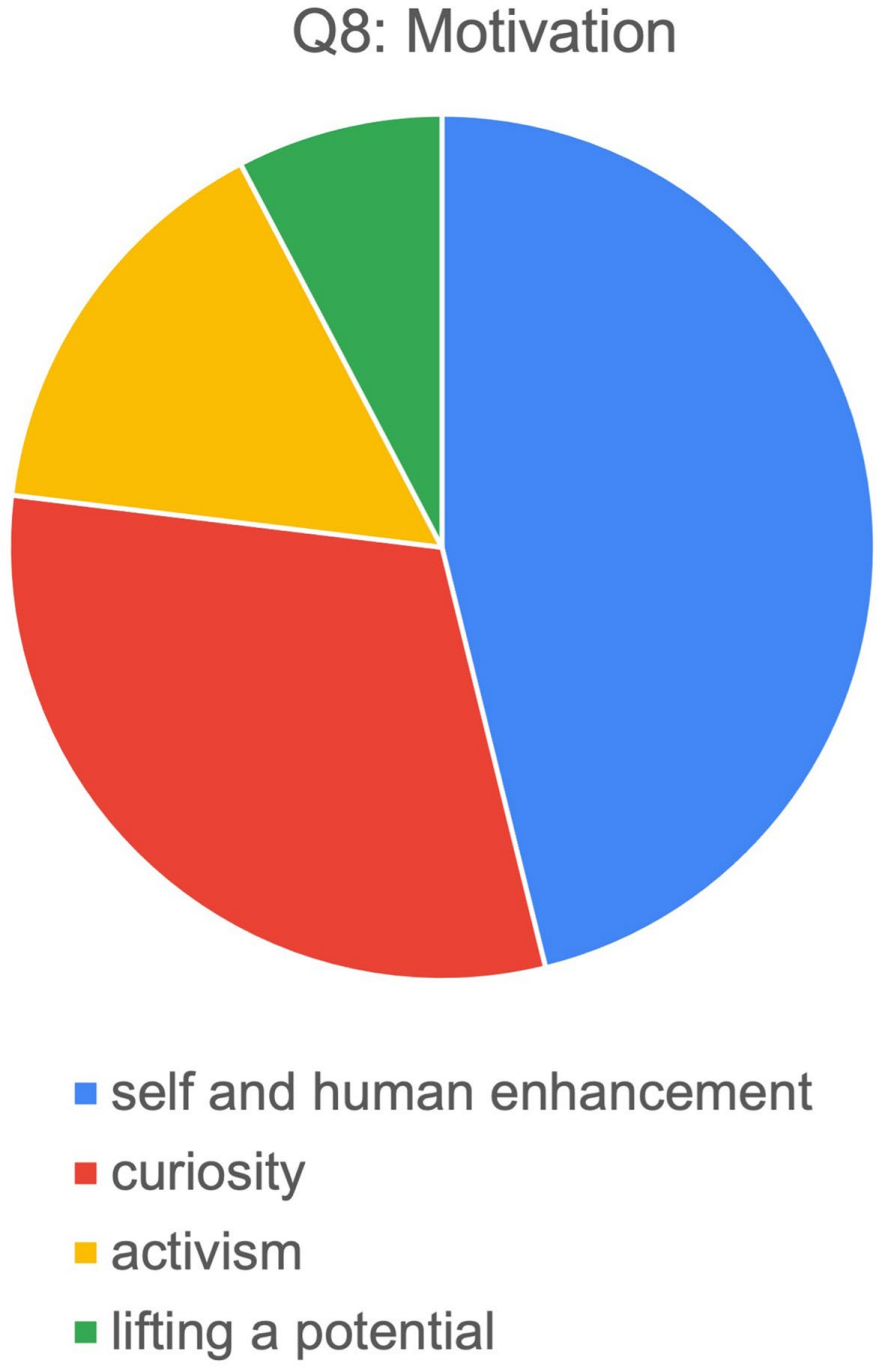
Motivation of respondents to start neurohacking (*n* = 13).

The key motivation (Q8) mentioned most frequently (by half of the respondents) is self-and/or human enhancement. <Rhi>, for instance, underlines the draw of NTs as “the concept of ‘self-technology’—regarding your mental and emotional makeup as something that can be approached with specific techniques,” while <Gil> and <Cal> are more drawn to the notions of human augmentation and enhancing the human condition, respectively (see Figure 1). Curiosity and a sense of adventure was also mentioned explicitly (by a third). Finally, two of the interview partners referred specifically to political or activist reasons besides human enhancement. <Pea>, as one example, discussed the principle of freedom of speech, which is seen by the neurohackers as linked to the freedom over one’s own body, referencing the beginning of the Internet with its promises to its current status quo and pondered, “how to build a new internet, using mesh networks, so if it is integrated into people’s biology, it cannot be taken away easily. Also, you can be an efficient data carrier, sitting in a cafe, uploads, downloads, you do not know who or what, you just carry an open network.” <Pea>

All of the interview partners except one indicated that their involvement with these technologies changed their views of the world and/or themselves, though in different and individual ways based on personal experience (Q9). These changes fell roughly under three groupings: social/outwards (six mentions), cerebral/inwards (five), and sensory/physical (three). Only one person, <Gil>, answered that they did not feel there had been much change in their views of the world, though they also mentioned that after having had an implant their acceptance of invasive procedures and body modifications had changed.

#### 3.2.3. Neuroabilities wish list

What kind of neuroabilities do our interview partners desire in near or far future, or in a utopian future? While our interview design originally addressed this in two separate questions dealing with, on the one hand, new neuroabilities they would like (Q10) and, on the other hand, what existent neuroabilities they might like to modify (Q11), our interview practice quickly showed us that many interview partners were either answering one or both questions and not necessarily differentiating between entirely new neuroabilities or existing neuroabilities they would modify. In total the number of desirable neuroabilities we collected from all 13 neurohackers came to 45, and, while many traits and abilities were cited only once or by one participant, they can be very easily grouped in three clusters: (1) *Senses* (20 instances), (2) *Memory/Cognition* (16 instances), and (3) *Body/Health* (9 instances) (see Table 3). One participant, <Cal>, discusses life extension and then mentions, “living longer by being uploaded in an atheist form of heaven.”

**Table 3:** What do neurohackers want to achieve or change?

Senses	Times mentioned	Body/Health	Times mentioned	Memory/Cognitive	Times mentioned
Visual	4	Expand nerve system	1	Brain optimization/mental performance/process faster	3
Audio	4	Fend off illnesses	1	Brain to brain communication	1
		Compensate aging	1	learning	1
Olfactory	1	Be uploaded beyond body	1	Think differently/new	1
Sense using magnetics	2	Cure Alzheimer	1	Perfect memory/recall	3
Other new senses	2	Cyborg dentures	1	Overcome trauma	1
Color	2	Sleep modulation	1	Better memory/abstract memory	2
Echolocation	1	Blood circulation as charger	1	Brain expand/ piggyback AI	2
See data like Molly Million	1	Control another’s body	1	Neuralink	2
Sense of orientation	1				
Senses to space	1				
Internet enabled senses	1				
Sum senses	20	Sum body/health	9	Sum memory/cognitive	16

#### 3.2.4. Being cyborgs?

This set of two questions sought to query whether the neurohackers identified as “cyborgs” (short for cybernetic organism) (Q13), and, since the term can be understood in different ways by different people and in different contexts, we asked our interview partners to define the term for us (Q12). The answers varied from very general definitions of technology integrated into one’s life or the merging of biology with technology; to very specific definitions, that discussed examples such as medical devices like pacemakers, cochlear implants, prosthetic limbs, or even vaccinations, to illustrate whether these examples fit into each neurohacker’s definition of a cyborg or not.

Non-invasive technologies that mediate or enhance human lives, such as glasses, wearables, or smart phones were only considered by three out of 13 interview partners as sufficient to be included under their definition of cyborg. Indeed, most neurohackers demanded a more intimate connection of technology and biology for someone to qualify as a cyborg in their eyes: Eight of 13 neurohackers defined that connection as synthetic and/or inorganic technology that is integrated into the body, breaking the skin barrier. Medical devices and procedures were specifically brought up by three of the neurohackers in regard to defining the term “cyborg,” and one of those three (<Vel>) further differentiated between people who “have a cybernetic dependence, because of medical issues” and people who “basically become cyborgs electively, without a medical need,” hinting at enhancement. Another aspect associated with the definition of “cyborg” included mentions of science fiction and extra abilities, brought up by three interviewees.^1^

When asked if they identified themselves as a “cyborg,” 11 out of the 13 neurohackers did so: some with a simple yes, others more emphatically, referencing their implants, peg legs etc. Very poignantly illuminating the very different definitions and meanings the term “cyborg” can take on, <Tao> argued that technology has already become such a part of human life that we are all now cyborgs.

The two neurohackers who did not identify as “cyborg” were < Gil> and < Nas>. The latter resisted defining the term “cyborg” and concluded that they considered themself a human being. The former felt that current implants can do too little and do not enhance enough, even while acknowledging that within the community the insertion of an implant serves as the barrier to formally be considered a “cyborg” and that they had been congratulated for becoming a cyborg upon receiving their implant.

#### 3.2.5. Impressions and visions

The first part of this interview section focused on what kind of impressions, associations and visions each neurohacker connects with NTs (Q14-16). We asked each interview partner to list five adjectives they associated with neurohacking (Q14). We counted a total of 52 attributive or descriptive quotation snippets, of which 44 (given by all 12 respondents) leaned positive, while only eight snippets (or 15%) given by four respondents could be considered as mixed or, in some instances, negative. The 52 quotations were clustered in six groups (see Table 4).

**Table 4:** Adjectives the respondents associated with neurohacking (each person could name up to 5) (*n* = 52).

Function	Times mentioned	Curiosity		Fun/Excitement		General positive terms		Mixed or negative terms		Future/Forward-looking	
Enhancing	3	Curious	4	Intriguing	2	Profound	1	Commercial	1	Pioneer	2
Tech positive	2	Experimental	3	Cool	1	Optimistic	1	Gray	1	Modern	1
Health	1	Learn	1	Fun	1	Social	1	Minor	1	Progressive	1
Therapeutic	1	Because we can	1	Interesting	1	Humane	1	Loose	1	Future-oriented	1
Enabling	1			Exciting	1	Potential	1	Negative use potential	1	New	1
Functionality	1			Stimulating	1	Worthy	1	Pyramid scheme	1	Cutting-edge	1
Performance	1			Risk friendly	1	Feminist	1	Scary	1		
				Brave	1	Intersectional	1	Violent	1		
						Anti-religious fanatics	1				
Sum function	10	Sum curiosity	9	Sum fun/excitement	9	Sum general positive terms	9	Sum mixed or negative terms	8	Sum future/forward-looking	**7**

Another question in this interview section concerned itself with what outcomes or potential the interviewees foresaw for NTs (Q15). All neurohackers could give more than one answer, and we received 27 answers from 12 respondents. Seven of those 12 respondents saw large potential positive outcomes for NTs in general and neurohacking specifically in the medical area and in applications that could contribute toward a better quality of life. Interview partners also mentioned generally improving people’s quality of life as well as specific fields and applications, such as geriatrics, bionic parts, and that people with hearing and visual impairments could benefit from NT applications. Five respondents saw potential in the areas of general and sensory enhancement and augmentation, either with general statements, such as “a significant potential to augment humans toward the next step” (<Gil>) or referring to upgrading, as well as enhancing humans, or with specific examples, such as “performance enhancement in athletics” (<Vel>) or “night vision.” Three interviewees highlighted the potential for communication, two specific examples including “brain-to-brain communication” (<Sal>) and “IoP, internet of people” (<Gil>). Three other respondents characterized the potential for NTs and neurohacking with descriptors relating to the theme of “exploration” as an aim in and of itself, including discussions of learning, different perspectives and enjoyment or pleasure derived from the exploration of that field and potential new senses.

Other references included mentions of military uses (twice), including one specific example by <Sal> positing a “body with different military machinery,” space exploration (twice), and general unspecified commercial opportunities (once). In this section, only two respondents voiced caution or denied any potential or outcome. The former argued there was a potential for harm, if the technologies and procedures were not regulated. The latter came from a respondent, <Nas>, who throughout their interview highlighted their disillusionment with NTs and neurohacking, arguing “It’s all hypothetical and a lot of it is bullshit, there is no functional feedback, no big outcome at this point in time.”

The final question in this section sought to explore whether interview participants identified shared visions in the neurohacking community and, if so, how they saw themselves in relation to these (Q16). All neurohackers stated that visions were shared either to a high degree (seven of twelve respondents) or to a partial degree (five of twelve), and no one answered that there were no shared visions. However, of those who mentioned that visions were shared in part, three explicitly highlighted the individualistic nature of the practice and practitioners with their own goals and motivations, leading two interviewees to the conclusion that sharing occurred in a more functional or practical dimension, e.g., “sharing ideas” (<Sal>) or exchanging thoughts on tinkering on online platforms (<Ari>). Since the neurohackers responded freely to our open-ended questions, we counted 13 quotes from 11 respondents^2^ when it came to what types of shared visions existed.

The common or shared visions all respondents mentioned can be clustered in two categories:

(1) *aimed inwards* (7 instances): self-improvement and exchanging ideas toward individual (own) goals, such as upgrading themselves and upgrading their brains, or controlling applications and equipment, and.

(2) *aimed outwards* (6): improving other people’s lives or working for a greater good (six instances).

### 3.3. Organizational structure and modus operandi

#### 3.3.1. Working together

The majority of those interviewed, 10 out of 13, reported that they are working together in groups, pooling their skills, experience, and knowledge (Q17). The first question to the 10 collaborative participants was about the way their group formed (Q18). Six people pointed to conferences or conventions where like-minded people meet and explicitly mentioned, for example, the BDYHAX convention (mentioned twice) and the DEFCON Hacking Conference. Another way to find collaborators is through exchange with manufacturers, said 3 respondents. The internet, in particular social media, Facebook biohacking and Reddit groups were also mentioned three times. Two people mentioned physical spaces of groups meeting offline, such as hackerspaces, the grinder hardware scene, groups experimenting with biology and chemistry and piercing shops. Only one person mentioned universities and hospitals as sources for finding collaborators.

When asked how the group changed over time (Q19), seven people explained that the group was growing. Of those who reported that the community was growing, <Gil> specifically said that the number of people with implanted chips is growing; <Nas>, who works at an NT company, said that since the NT industry is not yet fully defined and people from different backgrounds and industries work on different aspects, the community was still in the process of slowly forming.

The next question to the collaborative participants intended to find out whether they view their community as having shared or divergent aims (Q20). Half of the people (five) explained that they partly share the same aims and partly have divergent views. For instance, <Ari> mentioned the commonalities of the community were to provoke, to cross boundaries and to call on attention and <Vel> explained that everyone had their own aims but shared the same ethics. Four interviewees said that they share the same aims, while one person (<Tao>) commented that the community had divergent views—though that answer also detailed that these divergent views, skills, and visions were all brought to the table.

Whether or not they help each other with individual projects, the question regarding helping each other (Q21) was answered clearly in favor, with nine people saying yes, and only one person said no (the latter was <Tao>, founder of an NT startup). The second part of this question also asked whether hackers participated in collective projects, which eight answered in the positive, while two indicated they did not.

The next question (Q22) was: Where do you see similarities or differences to other networks/groups? Six interviewees answered that they think they are similar or rather similar, while three said they are different or rather different (one person did not comment). People who underlined similarities said, for example, that ethical and moral goals are the same, as well as social aspects, like hanging out together, or having a progressive attitude. Another person said that there are similarities, but that they are not meaningful. One of those who thought of them as different, <Pea>, mentioned that there were two types of groups, one was a start-up group with for-profit ambitions, while the other type of group consisted of those who “do it for the love of it.” Others also referred to either for-profit companies or non-profit hackerspaces. Another person who highlighted the differences, <Nas>, pointed out that details are often not shared and believed that sharing is limited because of intellectual property concerns.

#### 3.3.2. Practicalities: money, advice, and output

**Figure 2:**
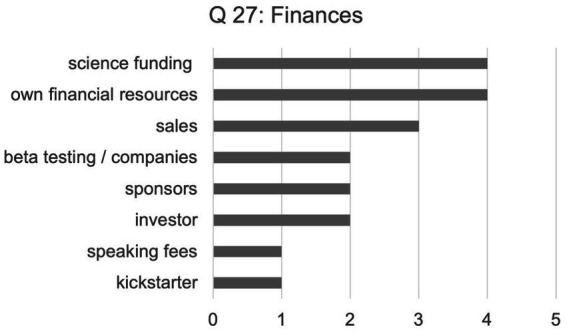
Source of financial support for neurohacking activities.

The next three questions were about practicalities, starting with: How do you finance your activities, are there any links to institutions or organizations? (Q27) The participants could name more than one financial source (*n* = 19), and the results showed a diverse range of finances, ranked by the number of instances recorded (see Figure 2).

Next, up was the question: Where do you go for input, advice or answers to your questions? (Q28) Again, more than one answer per person was possible (see Table 5). The one person who said that they have to learn things from scratch, mentioned that “surgeons had to practice on supermarket meat, since nobody had fired into the human nervous system before, even surgeons had to learn how to do it.”

**Table 5:** Source of input, advice, and answers to their questions (*n* = 21).

Source of advice	Times mentioned
The internet (social media groups, e.g., Facebook, What’s app groups or Biohack.me; but also manufacturers’ forums)	6
Their community (friends and other people from their network)	6
Specialists (including surgeons, university researchers, journalists, piercers, and tattoo artists)	6
Literature	2
Have to learn things from scratch	1
Sum	21

The final question (Q29) in this section was: What would you say is your output? Is there an audience, if so how would you say it’s made up? The target audience is mainly the public and the media (5 mentions), as well as their own community (4), researchers (2), companies or developers (1) and performers/magicians (1). Two people mentioned that there is no audience and that they do not share their content.

With regards to the content of their output, one person said it was novel applications to be shown to the community, another one aims to help others read and understand papers and be able to replicate experiments, yet another said the output is a fundamental critique of concepts of super individualistic enhancement without thinking about others. While one person stated that the output are NTs that help to make life easier, the same person acknowledged that the aim is also to show off some tricks and gags.

### 3.4. Ethics and risks

#### 3.4.1. Thoughts on ethics

When we asked whether ethics was a topic of interest or discussion in their practice and within their various NT groups and communities (Q30), only one of 12 respondents replied that they did not think ethics was a topic in the community. Of the 11 that responded that ethics was a topic, seven responded with qualifying remarks in their positive answers: they indicated that ethics did come up as a topic but peripherally. Several mentioned ethics as a topic at conventions, in online forums or during interviews, rather than during their NT practice or in their groups. Two interviewees mentioned that it was not a very central topic in regard to implants. One interviewee, <Vel>, even pointed out that ethics were a negative topic of discussion in their community, as something that prohibits NT practitioners from their practice or self-experimentation.

More specifically, ethical positions discussed in the past by the interviewees (Q31) centered around voluntariness (four answers), particularly around their freedom to modify their own body (e.g., with an implant) and that no one else should be able to tell them whether this should be permitted or not. The respondents, however, agreed that it was another situation when body modifications or human enhancement was done on other people, then a more stringent set of ethical rules and limitations was acceptable to them. The answers showed that the respondents saw ethics primarily as a justification for other people to ask them for permission, limit or prohibit their work. No one mentioned ethical debates as a way to identify useful ideas and applications. One participant mentioned that ethical behavior (of a company selling NT) could increase trust in consumers. Specific ethical issues mentioned were human enhancement, open access to technology, power structures limiting access to the technology such as access to capital, or existing gender disparities. At least two respondents commented indirectly on the changing nature of ethical and social norms. One (Ari) mentioned that while plastic surgery (e.g., breast implants) is seen as ethically acceptable, implants of functional devices are still mostly seen as unacceptable. Another (Tao) stated “For example removing an arm for a bionic one is considered unethical that might change in future, if it can be done easy and painless without the consequences.”

#### 3.4.2. Safety issues

How do neurohackers handle safety issues (Q32)? The most frequent answer (six respondents) was that they use materials and substances for implants that are well researched and understood, and that they share additional expertise about what works and what does not. Three commented on the importance of self-tests, that they either test a new product themselves before they give it to other people or eventually sell it to their customers, or that for example the piercer who injects an implant is doing it first on themself before implanting it into their customers. Two respondents highlighted that individual differences are significant. One of them brought up the example of the fine tuning of the electric stimulator for Parkinson patients, that a procedure is necessary to individually adjust the device based on the person’s specific requirements. The other one concluded that because of the individual differences, regulations that apply to everybody do not help.

When talking about safety, it was also asked which safety issues were identified (Q33). The most frequent response (five times) was about infections and healing after an implant has been surgically inserted. It was said that every surgery comes with the risk of an infection or other health problems, and that implants were no different. This was seen especially as an issue when the implants cannot be inserted through a standard medical procedure in a hospital, for example because regulatory or other professional rules prohibit doctors from carrying out such implant surgeries. The procedure then takes place at a piercing shop or in a hackerspace and participants stressed the importance of working in a sterile environment to avoid medical complications. The second most frequent safety issue (three respondents) was about hacking the implant or otherwise exploiting security weaknesses. This risk does not seem to be limited to devices developed by hackers, as one person mentioned that even a Parkinson stimulator could be hacked. One person mentioned that the implanted batteries could be a safety risk, for example when the battery bloats and breaks the protective coating, which could lead to the release of toxic compounds to the body. Two respondents stated that the law and regulations are a safety concern for them, as they want to avoid to (in)voluntarily breaking the law.

How do respondents assess reliability and quality of equipment (Q34)? Three neurohackers stated that they have to find out for themselves, either because they have professional expertise in the field, or they have ways to test the chips with their smartphone before implanting them. Three responses were made highlighting the importance of trust in the manufacturers, either through personal contact with them, through certificates or lifetime guarantees for implants. Two people mentioned that it is necessary to rely on standards in the production process of NT devices. One participant compared the efforts of developing an implant with a NASA mission, where every possible failure must be considered first and taken care of before the mission is launched (or the device implanted) as it is hard (or even impossible) to do repairs later. Two respondents were less optimistic, one stated that there is no way they could check the reliability and quality of equipment, while one commented that they cannot know if the device is reliable and of good quality.

#### 3.4.3. Trust and identified concerns

When asked if they had trust in the manufacturer’s claims (Q35) we got six who said yes, three who partly trust them and one who does not. Those who trust manufacturer’s claims gave different reasons for it, for example that they had themselves no way how to test the claims themselves and thus had to trust them, but also that they were in contact with the manufacturers and saw that they are responsible and transparent: one gave an example of a cooperative competition between competitors, where one manufacturer warned another one of a health risk of their product as such a risk would destroy the market for both of them. Different explanations were given by the three people who partly trusted the claims. One said that while there is trust in the reliability of the device, there is no trust in the security (see hacking in Q33) as there is a lack of open-source software to be checked. Another person said that while products from German or in general European manufacturers are to be trusted, this is less the case with manufacturers from the US. The third one claimed that while there is trust in the manufacturer’s claims, no one knows what happens when the device leaves the factory. Something could happen while the product is shipped to the customer. The person who answered that there is no trust, cited a lack of regulations and transparency as the reason.

The answers regarding their biggest (Q36b) and smallest concerns (Q36b) are summarized in Table 6.

**Table 6:** Overview of biggest and smallest concerns of respondents.

Biggest concern	Times mentioned	Smallest concern	Times mentioned
Immune response/infection (leading to death or amputation)	4	Infections (because of antibiotics)	2
Coating of the implant could breach (poisoning, e.g., battery)	3	Implant is injected too deep	1
General medical risk	2	Implant does not last long	1
Voltage injuries (tcds)	2	Chip fails and has to be replaced	1
Law	1	Implant is uncomfortable	1
No concerns	1	Credit card connected to the implant expires, rending the payment service and the implant without function	1
		What other people think	1
Sum biggest concern	13	Sum smallest concern	8

#### 3.4.4. Social consequences

The last question under the section ethics and risks was about social consequences of NT in general and in particular for disabled people (Q37). Regarding general consequences, the participants came up with a wide variety of answers (each answer mentioned once). They believed that NT, especially, implants, will create interest in other people, that it will cause people to adapt to intersectional thinking, that applications like interactive (glowing) tattoos will become a new fashion, that the fear of being hacked (i.e., that the implanted device will be hacked) will increase, that there will be an enormous potential for military applications, and that hate and opposition from the extreme religious right will further increase.

More consensus was observable regarding the consequences for disabled people, a sub-question which sprang from preliminary research and literature review. Nobody thought that NT will negatively affect the disabled, to the contrary, eight respondents believed that it will be positive for them. Explanations provided for the positive consequences for the disabled were manifold, including the belief that NT related body modification will cause people to overcome ableism because in the future people will understand that everyone is disabled (when they have no NT) so there will be no distinction between abled and disabled people, transforming the concept and self-concept of disability as such, both at the individual and social level. Another person mentioned that partially disabled people or people with autism could be provided with ways to better communicate with other people, another said that specific technical solutions (like IoT in conjunction with NT) will help the blind to better orient themselves, and still another foresaw that amputees will be have more functional prosthetic limbs that are not designed to fake a real limb but that highlight the prosthetic limb and their (additional) technical functionality (e.g., a prosthetic arm with a drone and other features).

### 3.5. Hackers’ future outlook

#### 3.5.1. Plans for the future

Four questions were asked about the interviewees ideas and plans for the foreseeable future. When asked which direction their work will take in the future (Q38), a variety of different answers were given (15, so some people mentioned more than one goal). Three interviewees stated that they want to contribute to the improvement and further development of existing products or devices, three planned to be active in educational activities, and three more commented that they wanted to contribute to sensory devices and related experiences. Two people mentioned payment solutions with implanted devices and two said that they wanted to wait until proper miniaturization for implants was available. One answer each was given for therapy and enhancement. One person did not provide an answer.

When asked about plans to commercialize their work (Q39), the neurohackers were split in two almost equally sized factions. While 6 had no plans or even opposed commercialization *per se*, 5 said that they had commercialization plans. Two of the “yes” answers stated that while they do not plan to commercialize a device, they do attempt to earn money selling their expertise through consulting companies or through speaking fees. One person was undecided, and one did not answer the question.

#### 3.5.2. Future tech wish list

When asked what they wished for future directions of hard-and software (Q40) the interviewees gave a variety of answers (respondents could give more than one answer) (see Table 7).

**Table 7:** Overview of what hard- and software respondents would like to see developed in the future.

Future tech wish list	
Miniaturization	4
Batteries/energy	3
Standardization	2
Medical monitoring	2
Accessibility/open/antidiscriminatory	2
Payment chips	2
Phone integration	1
Better documentation	1
Pain free and bloodless implantation	1
Flexible, bendable electronics	1
More applications	1
Improvements in the life cycle	1
Sum future tech wish list	21

The final question (Q41) asked participants to name the most probable next breakthrough. Here the interviewees came up with a wide variety of answers. In fact, only a breakthrough in payment systems was mentioned twice, all other answers were only recorded once, such as: open hardware, innovations based on Neuralink, better neuron-electron interaction, pain free implantation, a way to help or cure Alzheimer disease, applications in the field of smell and taste, improvements in stroke medicine, public/user acceptance, legal clarity in order to attract industry to invest more in the field, power supply, miniaturization, and improvements in the educational system.

The internalization of technology is seen by all as an ongoing process. Several interviewees believed that once the technological challenges such as energy supply, miniaturization, and increasing functionality have been sufficiently solved, the additional functionalities of these implanted devices will be appealing not only to a few pioneers but also to the mass market. The neurohackers speculated that further convergence of NT with AI, synthetic biology and other technologies will allow for an almost unlimited set of novel functionalities.

## 4. Discussion

The adoption and implementation of NTs outside academic and institutional frameworks has rarely been investigated from an academic point of view: the insights here provide a unique look at an overlooked group of everyday practitioners and potential drivers of NT and human enhancement. Drawing on all interviews, we seek to answer the five research questions stated in the introduction.

### 4.1. Neurohackers in the context of human enhancement

Neurotechnologies are clearly seen as key technologies to achieve human enhancement, but what if we look at it from the bottom up? Do neurohackers use NT with the goal to enhance their bodies, or are different contexts at play?

To tease out how practitioners view themselves, and in which ways their self-perception matches or differs, several questions in section 3.2 approached this topic from different angles, including motivation (Q8), the definition of the term cyborg and self-identification with that term (Q12–13), as well as associations connected to the practice of neurohacking (Q14). The key motivations that brought the neurohackers to their practices fell most commonly under the category of self-and/or human enhancement, though curiosity and a sense of adventure were also explicitly brought up. Correspondingly, the adjectives or associations they most connected with neurohacking fell under similar umbrella categories: associations with functions, such as “enhancing,” “therapeutic,” and “enabling”; descriptors connected to curiosity, such as “experimental” or “curious”; and words linked with fun, such as “exciting” or “cool.” Enhancement as a concept seems to be an inherent part of neurohacking practices, though other motivations and associations connected to excitement and exploration were also important to the hackers.

Enhancement is of course also linked to the concept of cyborgs, a term which can be applied as broadly as to most humans nowadays who use and are reliant on technology or as specifically as mechanical and artificial components integrated in the body (Clynes and Kline, 1960). Unsurprisingly, all but two of the neurohackers do self-identify as cyborgs. Yet how they define the term “cyborg” showcases some differences between how “human enhancement” is defined in general within the literature (UKMoD, 2021) and how enhancement in connection with the term “cyborg” is defined by neurohackers.

### 4.2. (Mostly) invasive enhancements for neurohackers

NTs can be applied either invasively and surgically, e.g., implants, or non-invasively, using, e.g., brain-machine-interfaces (BMI) or other wearables. Do neurohackers have a preference over these two forms? Does it have to be invasive to count as “real” neurohacking?

Defining the term cyborg (Q12), most of the neurohackers specified an intimate connection of body and technology to qualify as a cyborg, a connection incorporated in the body beneath the skin barrier (and three people specifically made mention of medical devices). Only three interviewees considered non-invasive technologies (such as wearables, phones etc.) as viable technologies in their definition of cyborgs. Noteworthy, of those three, two interviewees were part of an NT company, which had developed a semi-invasive product in the past and worked on developing wearables at the time the interviews took place. In most academic definitions of human enhancement cited in this text, an enhancement is not limited to invasive options, but also includes non-invasive options. The definition advanced by the SIENNA project for instance focuses on “science-based and/or technology-based interventions in or *on* the human body” (Jensen et al., 2018); emphasis added. The UK Ministry of Defence (in partnership with the German Bundeswehr) takes a long-term view of centuries of technology development to human augmentation, arguing similarly that human augmentation is independent of (im)permanence and (non-)invasiveness, but rather based on what it is used for (UKMoD, 2021).

Social events such as chip parties and conventions are part and parcel of the hacker subculture, and function as a space that provided many of the interviewees with the opportunity to form hacking groups (Q18). The appeal of implants and invasive devices also overlaps with body modification communities. But an implant may have to be removed for medical scans or procedures on the one hand or may simply grow weaker or stop functioning on the other hand. To some hackers this might represent a poignant dead weight, as, for instance, hacker-*cum*-reporter Adi Robertson describes at length (Robertson, 2017). Others might not be willing to give up what they perceive as a new sense—even if it involves surgical removal, left-over scar tissue, and an implant in a new spot. From our interviews one such example was <Vel>, who mentioned, while talking about motivation (Q8),

“With my magnetic implants, when they got rejected, I immediately got new ones. Life is just different without it, it’s like losing a sense, even if it’s just a small one, it is part of my existence. When I dream at night I have this magnetic sense, it is just part of me now, I guess.”

As noted, two participants involved in NT start-ups did mention wearables more frequently, owing to a larger market for such devices. Another hacker, <Gil>, points out, when speaking of which direction hacking work should go (Q38), there need to be “practical” applications that are significantly better than wearable options. Several of the hackers, discussing how technology should develop (Q40) and what the next big breakthrough is (Q41), point to miniaturization, batteries, and power supply issues. Several hackers make mention of minimally invasive, injectable technology in their answers. All of this indicates that, while implants represent a large and perhaps even essential part of the enhancement experience to hacking practitioners, they are well aware of both: current technological limits and issues, as well as the lack of appeal of significantly invasive technology to a general public. Still, it seems several hackers wish for a broader uptake of invasive NT, not so much rooted in specific functionalities of implants, but driven by a vision of humans becoming cyborgs (Est et al., 2014).

Outside of hacker and body modification circles, invasive measures (as opposed to wearables) are not necessarily viewed as an essential aspect or key quality of human augmentation. In fact, in its extensive paper on human augmentation, the UK Ministry of Defence notes: “Preparing to de-augment will be just as important as augmenting in the first place” (UKMoD, 2021, p. 67).

Written from a military perspective, this notion is certainly understandable, especially when it considers augmented soldiers and their return to civilian life. But the notion of *deaugmentation* is also poignant in the context of technology that may malfunction or stop working, and thus needs to be first surgically dug out before it can be repaired—or else remain functionless within the body.

A rather riveting example of malfunctioning and defunct implant technology was profiled recently, concerning the fate of patients with defunct retinal implants (Argus and Argus II) from company Second Sight (SSMP, 2023). When Second Sight announced its merger with a biopharmaceutical company, patients with their brain implant (Orion) debated an implant removal before funding runs out (Strickland and Harris, 2022).

### 4.3. Distinguishing between therapy and enhancement?

Human enhancement is frequently set in contrast to treatment or therapy, although the same technology might be used for both: the technology is used for improvement rather than for medical needs. While acknowledging that there are gray areas in the treatment/enhancement distinction, one author, for instance, maintains that he will use the distinction as it remains meaningful (DeGrazia, 2005, 263f). Similarly, the UK Ministry of Defence (and the German Bundeswehr) argue in their comprehensive publication that the definition of augmentation or enhancement is based on outcome (UKMoD, 2021), i.e., is the technology used to help overcome limitations? Or is it used to go beyond the capabilities of a human body (Agar, 2014)? Does that distinction make sense to neurohackers in practice? Is there a clear distinction between the two or is it a fuzzy transition?

The topic of medicine or therapy was brought up in answer to several questions, like Q15 which looks at the potential of NTs. Medical possibilities and quality of life were mentioned most in discussion by seven of 12 hackers, who answered that question. The answers stretched from very general mentions of “helping people” and “improving quality of life” to very specific mentions including “geriatrics,” “bionic parts,” and “people with disabilities.”

The topic also arose when it came to questions of future development (Q40). While many were concerned, as mentioned, with miniaturization or power supply, we also received two detailed responses discussing medical monitoring and drug delivery systems. When we asked hackers about the next big breakthrough (Q41), among the very varied answers, <Cal> brought up treating Alzheimer disease (and mentioned Elon Musk’s Neuralink project), while <Nas> spoke of stroke medicine and argued that the “pharma-industrial complex” had to undergo a fundamental change. A third hacker did not reference medicine explicitly but argued the next big breakthrough would see neurons growing together with electrodes.

We also asked neurohackers especially about what social consequences of NTs they foresaw in general and, as a sub-question, for disabled people particularly (Q37). Eight respondents argued that NTs help and would continue to help people with disabilities, e.g., that body modification can serve as a mode or tool of anti-ableism, that neurotechnologies could help people who are partially disabled or are affected by autism, that they could help the blind by providing sensory substitution, and that they could offer amputees a wide range of varied prosthetics. <Vel>, speaking of prosthetics, detailed they had friends with “amazing bionic limbs with drones attached and flashlights on them,” some of which these friends had created themselves.

Generally, the neurohackers indicated no potential negative consequences for persons with disabilities at all, though potential negative consequences and ethical dilemmas have been discussed and acknowledged in academic research. For instance, a reliance on neurotechnologies for disability management may inadvertently perpetuate societal stigmatization: emphasizing the need for technological interventions may well reinforce the notion that individuals with disabilities are inherently deficient and require constant augmentation to be considered “normal.” In turn, this may contribute to further marginalization of these individuals and undermine their autonomy in choosing whether or not to adopt these technologies (Aas and Wasserman, 2016).

To add another dimension to an already complex discussion, while the equitable access and ethical use of NTs were universally recognized as essential principles by all neurohackers, cultural differences did not play a significant role in our interviews and were not mentioned by the neurohackers beyond some positioning as a subculture distinct from the mainstream. However, different societies in general have varying perspectives on wellness, disability, and the importance of collective identities. These differences can affect not only the acceptance of neurotechnologies but also the ethical considerations surrounding their use (Salles et al., 2018).

Notably, beyond the one sub-question addressing impact on people with disabilities specifically, therapy and medical issues came up as a topic in answer to various other questions unprompted. Evidently, to most neurohackers the field of medicine is strongly linked to any future developments, breakthroughs, or goals that they hope NTs can achieve. This link also makes an appearance when it comes to development or funding. Four of the neurohackers we interviewed were or had been recipients of science funding (i.e., university-affiliated projects, Ph.D. programs etc.), of which one, <Sal>, emphasized that during their breakout project, an ambitious collaboration between engineering and medicine, “the medical side came with their own funding” which was significant (Q27).

Medical technologies and discoveries can serve as a basis for non-medical use cases. While the most reliable and accurate data on the brain comes from invasive brain-computer-interfaces (BCI) research with patients already subject to, e.g., DBS devices, that data can then be used for other purposes. Where invasive NTs are often associated with therapy and medicine, noninvasive NTs seem to be geared toward “enhancement” of “normal” bodies and disassociated from the medical field or from the realm of disabilities. Given this context, to draw a distinction between therapy and enhancement based on the technology itself seems disingenuous, if not outright impossible. Yet, is it useful to draw a distinction based on outcome, as suggested by the UK’s Ministry of Defence?

Much like people with a cochlear implant, who can use it to adjust to certain sound environments better than a person who does not have one, one of the interviewees explained a more complex relationship with his “headphone implants.” When asked about which neuroabilities they would like to modify (Q11), <Vel> mentioned that the implants were partly motivated by vision problems:

“I do have a problem with my vision, although I had a problem with one eye, my doctor told me I would go blind with the other eye, well not blind, but legally, I would not be able to drive. That was a lot of motivation to develop my headphone implants, I wanted to develop an echolocation system that does not bother others around me. My headphone implants did not end up working well for the echolocation.” <Vel>

<Vel> still had good eyesight starting the echolocation project, qualifying it as human enhancement, but <Vel> was preparing for a predicted loss of vision (which did not materialize), thus making the potential enhancement a therapeutic device. It seems hard to think of a better example to blur the boundaries between therapy and enhancement. Similarly, one of <Cal>’s answers to Q11 was fun cyborg dentures with radio and lights, a wish borne out of already needing dental replacements.

In short, neurohackers might simultaneously seek to manage a physical or psychological condition and to experiment with implants that open doors, pay bills or give them new senses.

### 4.4. Human enhancement (largely) about performance, optimization

In common definitions in literature, across research fields, human enhancement focuses on individual competitive advantages trying to increase the physical and cognitive performance of the human body beyond normal biological constraints, either temporarily or permanently (within transhumanism, the improvement of the human condition is contingent on these physical or cognitive improvements) (DeGrazia, 2005; Jensen et al., 2018; Raisamo et al., 2019; UKMoD, 2021). From the perspective of the military, enhancement and augmentation are necessarily tied to performance, function, and competition (Ienca et al., 2018; Ong, 2021; UKMoD, 2021). From a grassroots perspective, do neurohackers agree with this understanding (of a better, stronger, more optimized user) or do they implicitly or explicitly generate different interpretations of human enhancement?

How neurohackers view their extant or desired modifications, and how they relate to NTs comes up during various questions across the interviews.

In Q9 we ask whether hackers experienced a change of perception associated with NTs; the answers were clustered into three categories: social/outwards, cerebral/inwards, and sensory/physical. Answers in the first two (larger) categories could be associated with enhancement in a sense of optimization and efficiency.

For instance, <Sal> emphasized the potential of NT for therapeutic measures that could help disabled people, as well as its potential for enhancement, allowing communication by thought or interfacing with computers. <Nas> described seeing the limitations of humans as a result of being exposed to NT. <Vel> indicated their world view had become neurological (wondering whether representation should be neurological rather than political), an awareness which allows the opportunity for improving personally. <Cal> points out that neurohacking allowed them to be less afraid of death, to speculate less on prolonging life, since they would be informed and are part of a community that would be aware of any breakthroughs.

Answers in the smaller category of sensory changes touched on enhancement as exploration of new and additional senses. <Kee> described these changes, after using an antenna that allowed them to “hear” color, as “dramatic” and underlined that their sense of beauty had changed. <Tao> characterized their experience with the NorthSense as “enlightening,” and < Nol> described at length their interactions with magnetic fields in their everyday life. Similarly, a well-known neurohacker-*cum*-artist, Moon Ribas, developed Seismic Sense, feet implants which allow their user to sense earthquakes around the globe, and had the implant for 7 years (Ribas, 2019). In these cases, one could argue that these new senses represent a form of enhancement that are accompanied by perceptions of increased self-efficacy and/or a different self-image.

In Q10 and Q11 neurohackers discussed desired neuroabilities that were clustered in three categories: (1) *sensory enhancements* (20 instances) included (a) optimization/expansion (e.g., “improve visual cortex,” “seeing infrared or ultraviolet,” “better hearing”) and (b) exploration/curiosity (e.g., a “new sense of color,” “extend[ing] the senses to space,” a different “sense of orientation”). (2) *Abilities around memory and cognition* (16 instances) revolved around optimization (e.g., a “better” or “perfect memory,” “brain optimization,” “infinite recall,” “brain expansion”). (3) *Physical and health concerns* (9 instances) expressed wishes for optimization (e.g., “blood circulation as a charger” or “modulation of sleep […] without negative health effects”) and conventional transhumanist desires (e.g., “living longer by being uploaded” or “compensating the losses through aging”). Beyond the exception of new and exploratory senses and <Cal> who, as mentioned in section 4.3., wants fun cyborg dentures with radio and lights, these answers predominantly point to desires for self-optimization and enhancement-as-optimization.

Another question asked interviewees to list five adjectives associated with neurohacking (Q14). For a total of 52 descriptive snippets, overwhelmingly leaning positive, we found answers that could be clustered into four thematic groups and two other groups (section 3.2 for details). In terms of enhancement-as-optimization and enhancement-as-exploration, the descriptors across all groups match with answers we have reviewed and discussed in this and other sections above: while enhancement for the sake of convenience, function, performance, and optimization is predominantly associated with NTs (with an additional side nod to health and therapy), exploration and emotion remain strongly associated and connected to the topic.

The association of NTs with functionality/optimization, as well as with curiosity/exploration, is also reflected in answers to whether hackers felt there were shared visions within the neurohacking communities (Q16). Everyone felt visions were either highly or partially shared, and some of those common goals were very clearly targeted at self-improvement, performance, and optimization, such as “making life easier, control devices [with your implants]” or a comment that the “majority have an interest in more functional devices in the body.” The technologies are also viewed, as a few hackers mention, to help other people. <Pea> discusses in detail that some hackers were hacking for fun, while others wanted to “improve quality of life.” However, curiosity and emotion were also mentioned by several hackers, from a very general shared vision of “people enjoying having implants” (a side note to the mainstreaming of neurohacking discussed in section 4.5. below) to <Tao> passionately discussing curiosity as the one emotion that connected the community.

Across different questions within our interviews, it is apparent that NTs are innately connected to enhancement-as-optimization, in neurohacking practices—however they seem also, to a lesser degree but consistently, connected to non-utilitarian associations with curiosity, experimentation, and adventure.

### 4.5. Neurohackers contributing to “mainstreaming” neurotechnologies

“Neurotechnology often features as the harbinger of a future in which the body is transformed in a process of ever-increasing ‘technologization’” (Teunisse et al., 2019). The playful and experimental approach of neurohackers in conjunction with the creation and presentation of specific use cases of NT, raises the question whether neurohackers contribute to mainstreaming of the technology. Are neurohackers a kind of avant-garde, early adopters or pioneers who pave the way for a general trend to accept, adopt and deploy NT in the whole of society?

Our fifth research question sought to interrogate the role neurohackers play in mainstreaming neurotechnologies. To that end we wanted to go one step beyond the question of whether neurohackers are trailblazers (which in many cases they are, though sometimes they might blaze trails that are then not or only scarcely frequented).

When we asked those neurohackers who practice together with others about how their groups and communities formed (Q18) they mentioned for example conferences, conventions, and hackerspaces. In the context of this question, they also mentioned communication and exchange with manufacturers as a factor contributing to their group’s formation. In fact, the relations hackers have to manufacturers are in our opinion crucial to whether or not they have a role in “mainstreaming” NTs, and what that role might look like—these relations will be therefore discussed in further detail below.

The answers to Q20 and Q21 regarding shared aims and goals and whether they helped each other with individual projects showed that most hackers share (at least partially) the same goals and regarding group work and collective troubleshooting consequently indicate that collaboration and knowledge sharing are viewed as valued.

When it comes to (dis)similarities between hacker networks and groups (Q22) we found that the main difference is seen between non-profit and for-profit collectives. <Nas> who works for a startup, pointed out that there are “limits of sharing because of IP [intellectual property], details are often not shared,.” In terms of mainstreaming NT, in terms of reliability of NT products, companies are under higher scrutiny and need to adhere to regulations and standards in a way that do-it-yourself makers do not (Ienca et al., 2018).

Looking at the source of neurohackers input or advice (Q28) they named the internet, community/friends, and specialists. Regarding their audience (Q29) they predominantly mentioned the public, the media, and their own community. Exchange with academics or companies directly were rather scarce. “Mainstreaming” as a concept was mentioned by one, <Tao>, the CEO of a start-up, who indicated that, “[i]n the end, the audience should be anybody in 10 years, for now it is the curious people.”

Manufacturers made another appearance in the interviews, when we asked hackers how they assess equipment and its reliability (Q34). Three hackers (of 11) mentioned having trust in the manufacturers. <Nol>, for instance, referred to their personal exchange with a manufacturer and the role they took on as a beta tester. Similarly, another hacker, <Ari>, had also spoken of their function as a beta tester for a manufacturer, when we asked where they found their equipment (Q6). So at least indirectly, some neurohackers contribute to mainstreaming by becoming beta-testers for companies.

In this context it is important to note that neurohackers have trust in manufacturers’ claims (Q35). Several hackers highlighted that their trusted manufacturers communicated transparently (e.g., publishing their test results etc.) and that they had an ongoing dialogue with their communities. One hacker, <Vel>, pointed out in an anecdote that even potential rival producers would discuss quality issues with each other in order to not damage the interest and trust users have in the market at large.

Ethical issues are certainly also important for mainstreaming (Q30), and an overwhelming majority agreed on the importance of ethics in NT. However, many contributed remarks that indicated that ethics, while a topic, was not something connected to their NT practice, but rather a topic to be discussed at conventions or in interviews. When we asked them about ethical positions that they had discussed in the past (Q31), a good third of the answers focused on voluntariness and self-responsibility. Several of those respondents also highlighted they felt there was an imbalance of acceptance toward bodyhacking, especially when compared to accepted body modification such as plastic surgery. In general, ethics was discussed as a topic that might even serve to increase trust by consumers. It should also be noted that one respondent, <Nas>, was specifically hired to work for an NT startup as a strategist and to review potential ethical issues.

Beyond their positions on ethics, we also reviewed hackers’ perspectives on safety issues, concerns, and future directions. When we asked hackers what safety issues they identified (Q33), the most frequent response dealt with infections and healing, hacking and security exploitations. As a side note, two hackers also listed the law and regulations as a safety issue they have to be aware of, which reflected some of the answers discussing ethics as a prohibitive subject (Ienca et al., 2018). When we asked hackers how they handled safety (Q32), they responded that they used materials and substances that are well researched, and that additional expertise is shared. Several hackers also underlined the importance of self-tests and (again) working together collectively. Startup CEO <Tao> mentioned, for instance, that they are “the ‘first’ client of the company,” and highlighted again the importance of trust with the customers.

In terms of future directions, an initial question asked hackers about the potential of NTs (Q15), and they answered with various improvements, sectors and industries led by medical, but also including communication, the military, space, and commercial applications. In a later round of questions, we asked interviewees for their “wish list” in terms of future hard-and software (Q40), and the answers were very specific. A focus lay on what is seen as the largest two problem areas, which several hackers also identified as obstacles: miniaturization and power supply. NT implants must become much smaller, easier to implant, and useful to hackers and laypeople alike, or, as <Gil> put it succinctly in a very short answer, “compact, powerful, and pain free.”

Similarly, when we asked hackers to name the next breakthrough (Q41), they came up with many potential fields and applications across sectors. Here as well, miniaturization and power supply are raised as seminal issues. As <Vel> points out, neurohackers move in an arena of body modification that does not allow surgeons to implant them (as they are not medical devices), which is why, “[…] when things become injectable, they have the possibility for really mainstreaming and really getting into the public […].” <Gil>, who had also previously spoken of the necessity for pain-free and smaller implants, points to the importance for the whole field to find “‘practical’ applications that [are] significantly beneficial over wearables,” adding that that has been the most difficult exercise for the field. Referring to the perception of neuro-and bodyhacking from a larger public, <Gil> adds, “We must find a strong answer to convince people with negative thoughts.” Again, it seems that neurohackers see the mainstreaming of NT implants as a goal decoupled from their functional advantage they might (or not) entail.

It seems clear that our participants already contribute toward mainstreaming much beyond personal interest, individual projects, and self-experiments: they do this through continuous knowledge production and sharing, open communication lines with each other, strong relationships with manufacturers in a field that is small enough to remain permeable between knowledge sharing and commercialization. In fact, when we asked the hackers specifically whether they had plans to commercialize their work (Q39), just under half indicated they did.

Beyond the bold step of commercializing their hacking practices and in addition to it, “mainstreaming” has proven to be a topic of interest to most hackers we interviewed. Sometimes “mainstreaming” was brought up as part and parcel of larger social acceptance toward neuro-and bodyhackers, but often the topic is raised from a place of genuine interest, i.e., each hacker enjoys their modifications and practice to a degree that they would like to carry them out beyond their communities. However, the degree to which they are committed to neurohacking becomes very apparent throughout the course of each interview in general, and specifically perhaps when we asked them about their ideas and plans for the future: from developing further applications to exploring new areas such as sensory augmentation, to developing even smaller implants. Some of the hackers with academic background want to move forward into educational work, other hackers who identify as mostly consumers (as opposed to developers) are keen to stay active in the community and to carry their practice to other places and venues.

To our knowledge, this is the first academic survey focusing on neurohackers, highlighting their motivations, goals, tools, and practices. While they may be seen as a special interest niche community, their discourse and technical advancements do indeed represent a new perspective beyond academic and industrial interests. For example, in contrast to academia, neurohackers do not seem to spend too much time discussing ethical conditions or limitations when venturing from therapy to enhancement. Compared to industry, their ethos circles around open access and open-source technologies to allow everyone to access and use NT devices, with only a low to intermediate interest in making money with it. In this way, neurohackers function as pioneers both in terms of providing arguments *why* people might use NTs as well as how to access and deploy NTs.

### 4.6. Limitations

There might be a selection bias in our sample since we only contacted those neurohackers that were either visible online or that were recommended by our interview partners. This means that in principle we could have missed people who are either less visible (e.g., posting in non-English or non-German languages or in spaces specific to a particular language, country or culture; talking in replies rather than producing original posts…) or completely “offline.” (e.g., due to limited access to the internet; personal preference to not participate “out loud” or to attend offline spaces…). As mentioned in section 4.3, cultural differences were not significant in the interviews, though such differences may well be interesting to explore in future research. While we aimed to conduct online video interviews with all participants, 3 out of 13 insisted on providing written responses. There might be a slight difference in the answers provided depending on the interview style (online video or written), as an in-person video interview format lends itself to further conversations and probing questions.

## Data availability statement

The datasets presented in this article are not readily available because the data consists of responses from survey participants. Requests to access the datasets should be directed to schmidt@biofaction.com.

## Ethics statement

The studies involving humans were approved by Markus Schmidt and Camillo Meinhart (Biofaction KG). The studies were conducted in accordance with the local legislation and institutional requirements. The participants provided their written informed consent to participate in this study.

## Author contributions

GS and MS contributed to conception and design of the study. GS conducted the interviews and wrote the first draft of the manuscript. GS, SY, and MS carried out the qualitative analysis of the interviews. SY and MS prepared the second draft of the manuscript. All authors wrote sections of the manuscript and contributed to manuscript revision, read, and approved the submitted version.

## Footnotes

^1^In the flow of two different interviews we asked if the two interview partners thought getting a vaccine could make them cyborgs by altering their immune system. While one of them insisted that it would only work if the vaccine had an inorganic component, the other one referred to their exposure to the field of synthetic biology which has inspired them to compare genetic design and engineering with a form of small machines or nanobots. We also discussed the use of glasses with two interview partners, who both did not view that as a qualifying aspect for being a cyborg.

^2^One of the twelve respondents to Q16 did not list or define the shared visions they saw in the community.
