# Supplementary material for: Pioneering neurohackers: between egocentric human enhancement and altruistic sacrifice
Source: Front Neurosci. 2023 Oct 25;17:1188066. doi: 10.3389/fnins.2023.1188066 (PMC10631784; doi:10.3389/fnins.2023.1188066)
Supplement: Supplementary file 1 [file Table_1.DOCX]

Supplementary Material

Pioneering neurohackers: between egocentric human enhancement and altruistic sacrifice

Günter SEYFRIED*, Sandra YOUSSEF, Markus SCHMIDT

*** Correspondence:** Corresponding Author: [schmidt@biofaction.com](mailto:schmidt@biofaction.com)

# Supplementary Data

**Annex: interview questions guide**

**Intro & background**

**Q1:** Can you briefly tell us about yourself and what you do?

**Q2:** When and how did you start your "brainhacking" activities?

**Q3:** Which features or aspects resonated most with you? What was your access or entry point?

**Q4:** Do you see a connection to your education and occupation?

**Equipment & methods**

**Q5:** Can you tell us how and with what you do your neurohacking?

**Q6:** Where did you find the equipment and how did you decide on it?

**Q7:** Have you tinkered with it or otherwise modified it?

**Motivation & identity**

**Q8:** We’ve briefly touched on your access points before. To go a little further in-depth: What kind of motivational forces draw you to neurohacking?

**Q9:** Did neurohacking change your views on the world and/or yourself? In what ways?

**Q10:** Which neuroabilities do you want to gain that you don’t have?

**Q11:** Which neuroabilities (that you have) do you want to modify through your neurohacking activities?

**Q12:** We often hear the term cyborg in connection with hacking cultures. How do you define the term, how would you explain “cyborg” to someone who hasn’t heard the term before?

**Q13:** Do you consider yourself a cyborg? Do you know others that are?

**Q14:** What 5 adjectives would you connect or associate with your neurohacking? Why did you choose those?

**Q15:** What kind of outcome or potential do you see in these technologies?

**Q16:** Do you think there are sort of shared visions in the community? Would you say they’re more positive or more negative? Do you share these visions, too?

**Network & structure**

**Q17:** Are you brainhacking by yourself or is there a network, community, group, friends?

If answer is yes: continue with Q18-Q23, before jumping to Q27.

If answer is no: continue with Q24.

**Working with others:**

**Q18:** Can you tell us a little more? How did your partnerships or group form? How did you get to know others?

**Q19:** Did this group grow or change over time? And how do you connect?

**Q20:** What aims does your group have? Are they divergent, or are there aims you all share?

**Q21:** Do you help each other with individual projects? Do you work on collective projects?

**Q22:** Where do you see similarities or differences to other networks/groups? How about other interest- or hacker groups?

**Q23:** What else would you like to tell us about your group?

**Working alone:**

**Q24:** How does the process of hacking work for you – can you tell us a little more?

**Q25:** Where do you draw inspiration from, is there an art element to your work, a social element?

**Q26:** Is your work directed inwards or outwards?

**All:**

**Q27:** Can we talk about practicalities? How do you finance your activities, are there any links to institutions or organizations?

**Q28:** Where do you go for input, advice or answers to your questions?

**Q29:** What would you say is your output? Is there an audience, if so how would you say it’s made up?

**Ethics & risks**

**Q30:** How do you view ethics around neurohacking? Is ethics a topic that pops up and is discussed?

**Q31:** What positions are taken? It’s a very diverse field, so what kind of concerns could you identify? How do you handle them? How about other neurohackers?

**Q32:** How do you handle issues of safety in neurohacking?

**Q33:** What kind of safety issues could you identify?

**Q34:** How would you assess the reliability and quality of the equipment you work with?

**Q35:** How about your trust in manufacturers’ claims or how transparent manufacturers’ statements are? Can you test or check these claims?

**Q36:** What is your biggest and your smallest concern in regards to issues of safety?

**Q37:** What could be social consequences of your activities? And more specific: What could be social consequences for disabled people?

**Future outlook**

**Q38:** In which direction do you think your work will develop?

**Q39:** How about the economic aspects, any plans to commercialize it?

**Q40:** In what direction would you like to see hardware (and maybe software) developed?

**Q41:** What is the next big breakthrough you hope for and/or expect in your field?
